# An analysis of mitochondrial variation in cardiomyopathy patients from the 100,000 genomes cohort: m.4300A>G as a cause of genetically elusive hypertrophic cardiomyopathy

**DOI:** 10.1186/s40246-024-00702-9

**Published:** 2024-12-05

**Authors:** Luis R. Lopes, William L. Macken, Seth Du Preez, Huafrin Kotwal, Konstantinos Savvatis, Neha Sekhri, Saidi A. Mohiddin, Renata Kabiljo, Robert D. S. Pitceathly

**Affiliations:** 1 Institute of Cardiovascular Science, University College London, London, UK; 2 St. Bartholomew’s Hospital, Barts Heart Centre, London, UK; 3 Department of Neuromuscular Diseases, University College London Queen Square Institute of Neurology, London, UK; 4 NHS Highly Specialised Service for Rare Mitochondrial Disorders, Queen Square Centre for Neuromuscular Diseases, The National Hospital for Neurology and Neurosurgery, London, UK; 5 William Harvey Institute, Queen Mary University of London, London, UK; 6 NIHR University College London Hospitals Biomedical Research Centre, London, UK; 7 Centre for Heart Muscle Disease, Institute of Cardiovascular Science, University College London, London, UK

**Keywords:** Mitochondrial DNA, m.4300A>G, Hypertrophic cardiomyopathy, Whole Genome Sequencing

## Abstract

**Background:**

A significant proportion of cardiomyopathy patients remain genetically unsolved. Our aim was to use the large genomes cohort of the 100,000 genomes project (100KGP) to explore the proportion of potentially causal mitochondrial (mtDNA) variants in cardiomyopathy patients, particularly in genotype-elusive participants. The homoplasmic *MT-TI* 4300A>G is unusual in that it typically presents with a cardiac-only phenotype, but *MT-TI* is currently not part of the genes analysed for non-syndromic cardiomyopathies.

**Results:**

We analysed 1363 cardiomyopathy genomes from the 100KGP project (of which only 172 had been previously solved) to detect disease causing mtDNA variants. MitoHPC was used to call variants. For controls, 1329 random subjects not recruited for a cardiomyopathy diagnosis and not related to any participant in the cardiomyopathy cohort were selected. We have additionally compared the frequency of detected variants with published UK Biobank data. Pathogenicity annotations were assigned based on MitoMap. Four patients, all with a diagnosis of hypertrophic cardiomyopathy (HCM) and without a previously identified genetic cause from the 100KGP clinical-standard analysis, were found to harbour the pathogenic *MT-TI* m.4300A>G variant (0.6% of HCM cases without a diagnosis).

**Conclusion:**

These data support the inclusion of *MT-TI* in the initial genetic testing panel for (non-syndromic) HCM.

## Introduction

More than half of patients with cardiomyopathy have an elusive genetic cause [1]. The absence of a genetic explanation limits diagnostic certainty, personalized management, family screening, and family planning strategies.

Mitochondrial DNA (mtDNA) is a short multicopy molecule which is passed on exclusively via the maternal line. Variants can exist in every copy of mtDNA or in a mixed population with wild-type mtDNA (a status known as heteroplasmy). MtDNA is not routinely analysed during genetic testing for cardiomyopathies and is typically only included as a second tier test if certain clinical red-flags are present, including matrilineal inheritance, diabetes, deafness, neurological disease, or early onset disease with metabolic features e.g., lactic acidosis, regression and seizures [2]. However, pathogenic variants in mtDNA are a well described cause of cardiomyopathy, with cardiac involvement occurring in up to 40% of primary mitochondrial disease [2]. Though most mitochondrial cardiomyopathies present with co-existing extracardiac features, rare causes of cardiomyopathy, including the homoplasmic *MT-TI* m.4300A>G variant, may cause isolated cardiac disease without systemic involvement, meaning red flags prompting mtDNA analysis may be absent.

The 100,000 Genomes project (100KGP) was a UK Government initiative to accelerate the introduction of Whole Genome Sequencing (WGS) in rare disease and cancer diagnostics in UK’s National Health Service [3]. This project utilised a gene panel approach to analyse genes with an established link to the patient’s phenotype. Cardiomyopathy patients were enriched for gene-elusive cases. Though mtDNA is captured in WGS, appropriate bioinformatic pipelines are not routinely applied for mtDNA analysis, including in 100KGP [4].

Our aim was to use the large genomes cohort of 100KGP to explore the proportion of potentially causal mitochondrial variants in cardiomyopathy patients, particularly in genotype-elusive participants.

## Methods

From 1363 genomes of cardiomyopathy patients, only 172 had been previously solved by the standard/clinical approach. As described in more detail elsewhere [4], this approach involved the following steps: WGS (Illumina TruSeq, HiSeq 2500) via the 100KGP. Human Phenotype Ontology (HPO) terms extracted from notes and submitted at recruitment. Based on these, gene panels were applied, constructed via “PanelApp”, which classifies genes as “green” (diagnostic), “amber” (borderline evidence), and “red” (insufficient evidence). Only “green” genes were included in the clinical analysis. For further prioritisation, a clinical scientist reviewed all tier one (loss-of-function variants and *de novo* protein altering variants in virtual panels applied) and tier two (non-loss-of-function protein altering variants in virtual panels applied) variants. “Likely pathogenic” or “pathogenic” variants were confirmed with Sanger and reported; otherwise a report stating there were “no primary findings” was released.

For our analysis, the bioinformatics pipeline, MitoHPC (https://github.com/ArkingLab/MitoHPC) was used to call mtDNA variants in 1363 genomes of cardiomyopathy patients. MitoHPC’s key feature is that it constructs a consensus mitochondrial sequence for each sample, and calls heteroplasmies against an individual unique mitochondrial genome, which greatly improves heteroplasmy estimates. MitoHPC performs two iterations of variant calling using mutect2; firstly to identify major alleles, or homoplasmies, which are used to construct the consensus mitochondrial sequence, and secondly to call heteroplasmic variants. As MitoHPC also provides various quality metrics at both sample and variant levels (e.g. mitochondrial copy number estimation, haplogroup determination, sequencing coverage statistics), we have limited our analysis to samples that are not labelled as suspicious by MitoHPC. Each reported variant passed the variant quality filter. Resultant mtDNA variants were reviewed by a clinician and only those with a heteroplasmy level of > 10% and a plausible link to the phenotype were analysed further.

For the control population, we have retrieved via the Genomics England LabKey application all Rare Disease participants with Participant Type ‘rd_relative’ and Affection Status ‘Unaffected’. We then removed participants recruited as relatives of subjects from the cardiomyopathy cohort. We randomly selected from the resulting table (using ‘head’ function in R on the unsorted table with unique participants) samples to match in numbers our cardiomyopathy cohort. From the resulting dataset, we have further removed those with HPO terms “mitral valve prolapse”, “palpitations”, ”syncope” and “prolonged QTc interval”. IDs of all cases and controls used are accessible to registered users of Genomics England Research environment, and can be found in directory: /re_gecip/shared_all_GeCIPs/4300/.

This produced a set of 1329 controls. We have additionally compared the frequency of detected variants with published UK Biobank (UKB) data [5].

Pathogenicity annotations were assigned based on the MitoMap database (http://www.mitomap.org/), which curates mtDNA variants based on their link to disease.

## Results

**Fig. 1:**
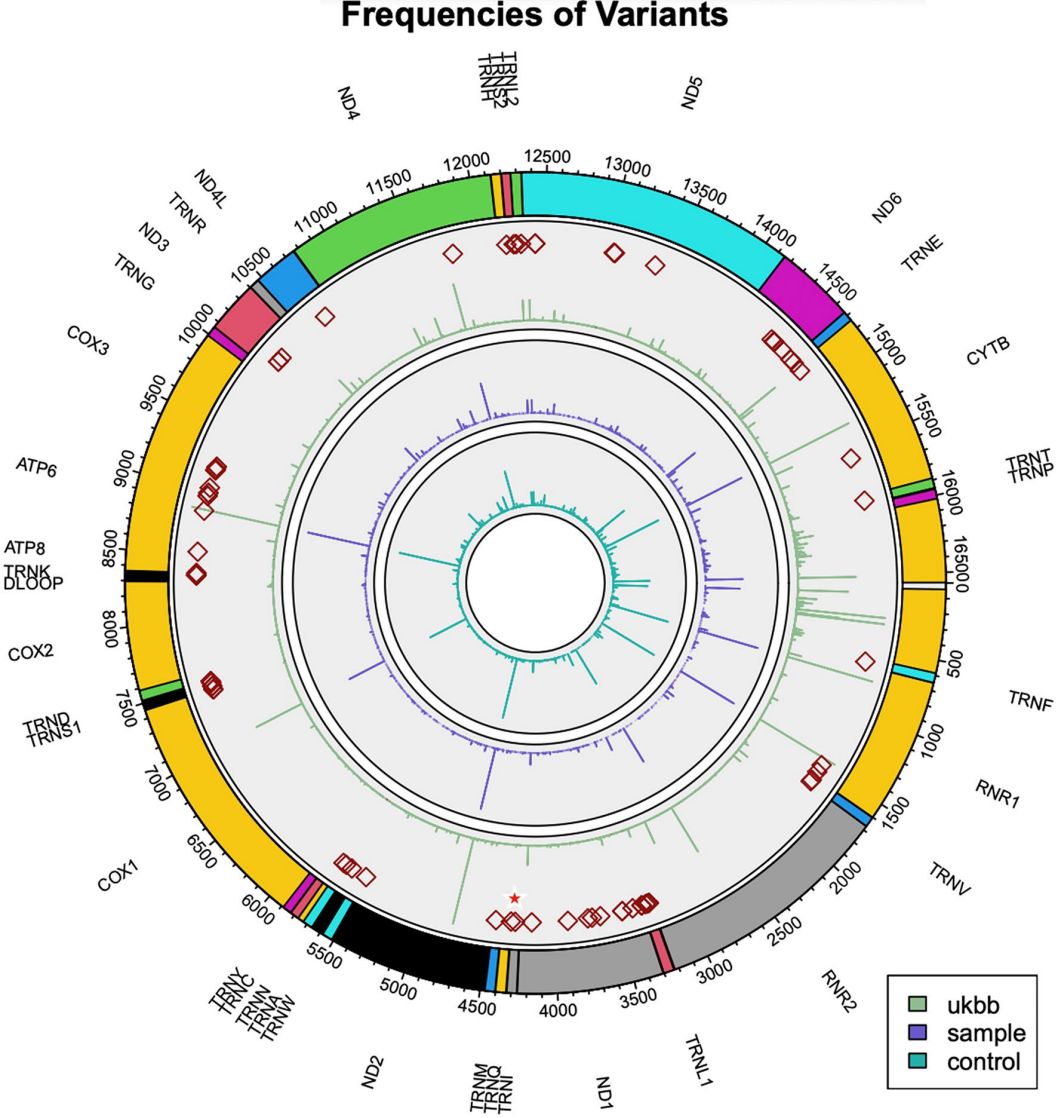
Frequencies of variants detected by MitoHPC in the control samples (the most inner circle, turquoise), in the cardiomyopathy samples (the circle in the middle, purple) and in the UKBiobank population (the outmost circle, green). Red squares in the outmost circle mark positions of confirmed pathogenic variants in MitoMap (https://www.mitomap.org/foswiki/bin/view/MITOMAP/ConfirmedMutations) that are present in the UKBiobank, none of which are associated with cardiomyopathy. Red star marks the position of m.4300A>G

Figure 1 shows frequencies of variants detected by MitoHPC in the control samples (the most inner circle, turquoise), in the cardiomyopathy samples (the circle in the middle, purple) and in the UK Biobank population (the outmost circle, green) [5].

From a total of 796 hypertrophic cardiomyopathy patients, only 112 had received a genetic diagnosis from prior 100KGP analyses. Four patients, all with a diagnosis of hypertrophic cardiomyopathy (HCM) and without a previously identified genetic cause from clinical tiers 100KGP analysis, were found to harbour the m.4300A>G variant (0.6% of HCM cases without a diagnosis). None of the individuals in the control group had the m.4300A>G variant. While a small number of additional likely pathogenic/pathogenic mtDNA variants were detected in cardiomyopathy cases, they have low penetrance and we consider these incidental findings without contribution to the phenotype. All were homoplasmic, apart from one (m.11778G>A, 32% heteroplasmy). Three individuals had variants that predispose to aminoglycoside-induced deafness (m.1555A>G x 2, m.1494C>T x 1). Four individuals carried Leber Hereditary Optic Neuropathy (LHON) risk variants (m.3460G>A x 2, m.10663T>C x 1, and m.11778G>A x 1). None of these variants have an established cardiac phenotype; although some have postulated a risk of HCM in LHON, this link remains unsubstantiated.

## Discussion

The homoplasmic *MT-TI* 4300A>G is unusual in that it typically presents with a cardiac-only phenotype (i.e., HCM in the absence of systemic/neurological features) [6]. Notably this condition exhibits variable expressivity and penetrance, and as such the typical matrilineal inheritance pattern associated mtDNA variants may be not be obvious to clinicians. The *MT-TI* gene encodes the mitochondrial tRNA for isoleucine. Interestingly, the penetrance of certain homoplasmic mitochondrial tRNA variants are thought to be influenced by additional variants in nuclear genes which play a role in tRNA biology, though such a link has not to date been established for m.4300A>G.

## Conclusion

With the future availability of WGS at clinical level, mtDNA will be analysed simultaneously with known nuclear causal genes. At the moment, *MT-TI* and other mtDNA genes are only included in panels testing for “maternally inherited cardiomyopathy” or syndromic disease. Our data support the inclusion of *MT-TI* as part of the initial genetic testing panels for (isolated/non-syndromic) HCM, as this will establish causality in a subset of hitherto genetically-elusive cases where maternal inheritance is not obvious or other extra-cardiac features are not present, with significant implications for the proband and family.

## Data Availability

The datasets analysed during the current study (primary data from the 100,000 Genomes Project) are subject to controlled access as they contain clinical information and the study’s ethical approval dictates that all data access and analysis must occur within the designated secure access environment. No data can be copied and removed from that environment without being subject to oversight from the study to ensure patient anonymity and data security. Data can be accessed worldwide by registered researchers who become members of a relevant Genomics-England Clinical Interpretation Partnership domain. All other data supporting the findings described in this paper are available from the corresponding authors upon reasonable request.
